# The American Society of Breast Surgeons and Quality Payment Programs: Ranking, Defining, and Benchmarking More Than 1 Million Patient Quality Measure Encounters

**DOI:** 10.1245/s10434-017-5940-1

**Published:** 2017-08-01

**Authors:** Jeffrey Landercasper, Lisa Bailey, Robert Buras, Ed Clifford, Amy C. Degnim, Leila Thanasoulis, Oluwadamilola M. Fayanju, Judy A. Tjoe, Roshni Rao

**Affiliations:** 1 Gundersen Health System, La Crosse, WI USA; 2 Bay Area Breast Surgeons, Inc., Oakland, CA USA; 3 Anne Arundel Medical Center, Annapolis, MD USA; 4 Baylor Scott and White Healthcare, Dallas, TX USA; 5 Mayo Clinic, Rochester, MN USA; 6 New York Community Hospital of Brooklyn, Brooklyn, NY USA; 7 Duke University, Durham, NC USA; 8 Aurora Health Care, Milwaukee, WI USA; 9 University of Texas Southwestern Medical Center, Dallas, TX USA

## Abstract

**Background:**

To identify and remediate gaps in the quality of surgical care, the American Society of Breast Surgeons (ASBrS) developed surgeon-specific quality measures (QMs), built a patient registry, and nominated itself to become a Center for Medicare and Medicaid Services (CMS) Qualified Clinical Data Registry (QCDR), thereby linking surgical performance to potential reimbursement and public reporting. This report provides a summary of the program development.

**Methods:**

Using a modified Delphi process, more than 100 measures of care quality were ranked. In compliance with CMS rules, selected QMs were specified with inclusion, exclusion, and exception criteria, then incorporated into an electronic patient registry. After surgeons entered QM data into the registry, the ASBrS provided real-time peer performance comparisons.

**Results:**

After ranking, 9 of 144 measures of quality were chosen, submitted, and subsequently accepted by CMS as a QCDR in 2014. The measures selected were diagnosis of cancer by needle biopsy, surgical-site infection, mastectomy reoperation rate, and appropriateness of specimen imaging, intraoperative specimen orientation, sentinel node use, hereditary assessment, antibiotic choice, and antibiotic duration. More than 1 million patient-measure encounters were captured from 2010 to 2015. Benchmarking functionality with peer performance comparison was successful. In 2016, the ASBrS provided public transparency on its website for the 2015 performance reported by our surgeon participants.

**Conclusions:**

In an effort to improve quality of care and to participate in CMS quality payment programs, the ASBrS defined QMs, tracked compliance, provided benchmarking, and reported breast-specific QMs to the public.

For more than two decades, strong evidence has indicated variation in the quality of cancer care in the United States.^1^–^19^ As a result, measurements and audits are necessary to search for gaps in the quality of care. Toward this end, multiple professional organizations have developed condition-specific quality measures (QMs) to assess the clinical performance surrounding the patient-provider encounter.

Quantification of performance can identify variation and opportunities for improvement. If performance assessment is followed by performance comparison among peers (i.e., benchmarking) coupled with transparency among providers, physicians who find themselves in the lower tiers of performance can be motivated to improve, ultimately yielding better overall care at the population level, a phenomenon that recently has been reviewed and demonstrated by several programs.^20^–^26^

This report aims to describe how the American Society of Breast Surgeons (ASBrS) ranked and defined measures of quality of care and subsequently provided benchmarking functionality for its members to compare their performances with each other. By separate investigations, the actual performance demonstrated by our ASBrS membership for compliance with nine breast surgeon-specific QMs are reported.

Founded in 1995, the ASBrS is a young organization. Yet, within 20 years, membership has grown to more than 3000 members from more than 50 countries. A decade ago, the Mastery of Breast Surgery Program (referred to as “Mastery” in this report) was created as a patient registry to collect quality measurement data for its members.^27^

Past President Eric Whitacre, who actually programmed Mastery’s original electronic patient registry with his son Thomas, understood that “quality measures, in their mature form, did not merely serve as a yardstick of performance, but were a mechanism to help improve quality.”^28^,^29^ Armed with this understanding, the ASBrS integrated benchmarking functionality into Mastery, thus aligning the organization with the contemporary principles of optimizing cancer care quality as described by policy stakeholders.^2^,^19^,^25^,^30^

In 2010, Mastery was accepted as a Center for Medicaid and Medicare Services (CMS) Physicians Qualified Reporting Service (PQRS) and then as a Qualified Clinical Data Registry (QCDR) in 2014, linking provider performance to government reimbursement and public reporting.^31^ Surgeons who successfully participated in Mastery in 2016 will avoid the 2018 CMS “payment adjustment” (2% penalty), a further step toward incentivizing performance improvement in tangible ways.

## Methods

### Institutional Review Board

De-identified QM data were obtained with permission from the ASBrS for the years 2011–2015. The Institutional Review Board (IRB) of the Gundersen Health System deemed the study was not human subjects’ research. The need for IRB approval was waived.

### Choosing, Defining, and Vetting QM

From 2009 to 2016, the Patient Safety and Quality Committee (PSQC) of the ASBrS solicited QM domains from its members and reviewed those of other professional organizations.^32^–^39^ As a result, as early as 2010, a list of more than 100 domains of quality had been collected, covering all the categories of the Donabedian trilogy (structure, process, and outcomes) and the National Quality Strategy (safety, effectiveness, efficiency, population health, care communication/coordination, patient-centered experience).^40^,^41^ By 2013, a list of 144 measures underwent three rounds of modified Delphi process ranking by eight members of the PSQC, using a RAND/UCLA Appropriateness Methodology, which replicated an American College of Surgeons effort to rank melanoma measures and was consistent with the National Quality Forum’s guide to QM development^42^,^43^ (Tables 1, 2). During the ranking, quality domains were assigned a score of 1 (not valid) to 9 (valid), with a score of 5 denoting uncertain/equivocal validity. After each round of ranking, the results were discussed within the PSQC by email and phone conferences. At this time, arguments were presented for and against a QM and its rank. A QM was deemed valid if 90% of the rankings were in the range of seven to nine.

**Table 1:** Instructions of the American Society of Breast Surgeons for ranking of quality measure domains

*Ranking* 42,43
1. [Evaluate the quality domains] for appropriateness (median ranking) and agreement (dispersion of rankings) to generate quality indicators
2. A measure [will be] considered valid if adherence with this measure is critical to provide quality care to patients with [breast cancer], regardless of cost or feasibility of implementation. Not providing the level of care addressed in the measure would be considered a breach in practice and an indication of unacceptable care
3. Validity rankings are based on the panelists’ own personal judgments and not on what they thought other experts believed
4. The measures should apply to the average patient who presents to the average physician at an average hospital
*Importance criteria* 57
1. Variation of care
2. Feasibility of measurement, without undue burden
3. Usability for accountability [public transparency or quality payment programs]
4. Applicability for quality improvement activity
*Scoring criteria* 42,43
1 = not valid
5 = uncertain/equivocal validity
9 = valid

Verbatim instructions from an American College of Surgeons ranking study^43^

**Table 2:** Hierarchy of quality domains for breast surgeons after the 3rd round of modified Delphi ranking

Quality domain	Median score^a^	Validity^b^	Agreement^c^
*Patients receiving diagnosis of cancer by needle biopsy*	9	Yes	Agreement
Patients undergoing a formal patient-side-site-procedure verification procedure in the operating room	9	No	Agreement
*Percentage of cancer patients with orientation of lumpectomy specimen*	9	Yes	Agreement
*Clinical stages 1 and 2 node-negative patients offered sentinel lymph node (SLN) surgery*	9	Yes	Agreement
Mastectomy patients with ≥4 positive nodes referred to radiation oncologist	9	Yes	Agreement
Stages 1, 2, and 3 patients undergoing initial breast cancer surgery with documentation of ER, PR receptor status	9	No	Agreement
Stage 1, 2, and 3 undergoing initial breast cancer surgery with documentation of HER2 neu status	9	No	Agreement
Breast conservation therapy (BCT) patients referred to radiation oncology	9	Yes	Agreement
Percentage of patients undergoing neoadjuvant therapy before planned breast conservation surgery (BCS) who have imaging marker clip placed in breast	9	Yes	Agreement
*Percentage of patients undergoing lumpectomy for non-palpable cancer with specimen imaging performed*	9	Yes	Agreement
Patients with concordance assessment (testing) of Exam-Imaging-Path by care provider	9	No	Agreement
Patients undergoing breast cancer surgery with final path report indicating largest single tumor size	8.5	No	Agreement
Patient’s compliant with National Quality Forum Quality Measures (NQF QM) for endocrine therapy in hormonal receptor positive patients	8.5	Yes	Agreement
Trastuzumab is considered or administered within 4 months (120 days) after diagnosis for stage 1, 2, or 3 breast cancer that is HER2-positive	8.5	No	Agreement
Documentation of mastectomy patients offered referral to plastic surgery	8.5	Yes	Agreement
Documentation of eligibility of BCT and eligible patients offered BCT	8.5	Yes	Agreement
Patients with documentation of patient options for treatment regardless of procedure type	8.5	Yes	Agreement
Percentage of patients undergoing BCT with a final ink-negative margin, regardless of number of operations	8.5	No	Agreement
Patients with adequate history by care provider	8	No	Agreement
Patients with documentation of postoperative cancer staging (AJCC)	8	Yes	Agreement
Patient’s compliant with NQF QM for radiation after lumpectomy	8	No	Agreement
Patients with documentation preoperative (pretreatment) AJCC clinical staging	8	Yes	Agreement
NCCN compliance with radiation guidelines	8	No	Agreement
Mastectomy patients receiving preoperative antibiotics	8	Yes	Agreement
Patients with NCCN guideline compliant care for “high risk lesions” identified on needle biopsy (ADH, ALH, FEA, LCIS, papillary lesion, radial scar, mucin-containing lesion)	8	No	Agreement
Patients with NCCN guidelines compliant care for diagnostic evaluation of breast lump	8	No	Agreement
Patients with NCCN compliance for postoperative lab imaging, biomarkers in stages 0, 1, and 2 patients	8	No	Agreement
NCCN guideline compliance for genetic testing among patients with newly diagnosed breast cancer	8	No	Agreement
NCCN guideline compliance for genetics assessment/referral among patients with newly diagnosed breast cancer	8	No	Agreement
Patients with adequate examination by care provider	7.5	No	Agreement
Patients with final pathologic size ≥ stage 1 T1cN0M0 who have documentation of discussion regarding adjuvant treatment	7.5	Yes	Agreement
Documentation of reason why patient is not eligible for BCT	7.5	No	Indeterminant
Patients with adequate review of imagining by care provider	7.5	No	Indeterminant
Patients with inflammatory or locally advanced breast cancer who undergo neoadjuvant treatment before surgery	7.5	No	Agreement
High-risk patients with estimated lifetime risk >20% offered screening MRI	7.5	No	Indeterminant
NCCN compliance for medical oncology recommendations	7.5	No	Indeterminant
Risk adjusted re-excision lumpectomy rate after breast-conserving therapy	7.5	Yes	Agreement
NCCN guideline compliance for inflammatory breast cancer	7.5	No	Indeterminant
NCCN guideline compliance for breast cancer in pregnancy	7.5	No	Indeterminant
Patients with predicted estimate of BRCA mutation >10% offered BRCA testing	7.5	No	Indeterminant
High-risk patients (no known cancer) with documentation of risk-reduction counseling	7.5	No	Indeterminant
NCCN guideline compliance for inadequate margins requiring re-excision in BCS patients	7.5	No	Agreement
Patients receiving antibiotics within 1 h before surgery	7	Yes	Agreement
*Patients receiving a first- or second-generation cephalosporin before incision*	7	Yes	Agreement
*Patients with discontinuations of antibiotics within 24 h after surgery*	7	Yes	Agreement
Patients with Surgical Care Improvement Project (SCIP) antibiotic measure compliance (includes all 3 measures above)	7	Yes	Agreement
*Patients with breast cancer with documentation of risk assessment for germline mutation*	7	No	Indeterminant
Patients compliant with SCIP DVT/PE prophylaxis recommendations	7	No	Indeterminant
Patients ≤50 years with newly diagnosed breast cancer offered genetic testing	7	Yes	Agreement
Patients presented to interdisciplinary tumor board (real or virtual) at any time	7	No	Agreement
Patients compliant with NQF QM for chemotherapy in hormonal receptor-negative patients	7	No	Indeterminant
Surgical-site infection rate (mastectomy patients)	7	No	Indeterminant
Percentage of patients entered into a patient registry to identify patient complications and cancer outcomes	7	No	Indeterminant
*One-step surgery success rate stratified by type of operation (mastectomy)*	7	No	Indeterminant
Sentinel lymph node identification rate (%) in breast cancer surgery	7	Yes	Agreement
Cosmetic score (measure of cosmesis) after BCS (patient self-assessment with Harvard score)	7	No	Indeterminant
Time (business days) from diagnostic evaluation to needle biopsy	7	No	Indeterminant
Time (business days) from needle biopsy path report to surgical appointment	7	No	Indeterminant
Surgical-site infection rate (mastectomy plus plastic surgery patients)	7	No	Indeterminant
Ipsilateral breast tumor recurrence (IBTR)	7	No	Indeterminant
Percentage of patients undergoing lumpectomy for non-palpable cancer with two-view specimen imaging performed	7	No	Indeterminant
Percentage of compliance with ASBrS or ACR annotation of ultrasound (US) images	7	No	Indeterminant
Percentage of compliance with ASBrS or ACR recommendations for US reports	7	No	Indeterminant
Percentage of compliance with ASBrS or ACR recommendations for US needle biopsy reports	7	No	Indeterminant
Compliance with ASBrS or ACR recommendations for US needle biopsy reports	7	No	Indeterminant
NCCN guideline compliance for pre-op lab and imaging in clinical stages 0, 1, and 2 patients with cancer	7	No	Indeterminant
Patients with preoperative needle biopsy proven axillary node who do not undergo sentinel node procedure	7	No	Indeterminant
Local regional recurrence	7	No	Indeterminant
Patients age ≥70 years, hormone receptor positive, with invasive cancer offered endocrine therapy instead of radiation (documentation)	7	No	Indeterminant
Disease-free survival	6.5	No	Indeterminant
Time business days from new breast cancer to office appointment	6.5	No	Indeterminant
Patients with predicted estimate of BRCA mutation >10% who are tested	6.5	No	Indeterminant
Time business days from needle biopsy path report of cancer to surgical operation	6.5	No	Indeterminant
Time business days from abnormal screening mammography to diagnostic evaluation	6.5	No	Indeterminant
Percentage of cancer patients entered into a quality audit (any type: institutional, personal case log, regional, national)	6.5	No	Indeterminant
Time business days from new breast symptom to office appointment	6.5	No	Indeterminant
Patients with benign breast disease with documentation of risk assessment for cancer	6.5	No	Indeterminant
Percentage of patients with partial breast irradiation after lumpectomy who are compliant with “ASBrS guidelines for eligibility”	6.5	No	Indeterminant
Percentage of patients with partial breast irradiation after lumpectomy who are compliant with “ASTRO guidelines for eligibility”	6.5	No	Indeterminant
Number of breast-specific CMEs per year	6.5	No	Indeterminant
NCCN compliance for SLN surgery in stage 0 DCIS	6.5	No	Indeterminant
Skin flap necrosis rate after mastectomy stratified by type of mastectomy reconstruction, type of reconstruction	6.5	No	Indeterminant
Overall survival	6	No	Indeterminant
Ratio of malignant-to-benign minimally invasive breast biopsies	6	No	Indeterminant
*Surgical-site infection rate (all patients)*	6	No	Indeterminant
Surgeon US (2 × 2 test table performance) (sensitivity, specificity, PPV, NPV) for surgeons performing diagnostic breast evaluation with imaging	6	No	Indeterminant
NCCN guideline compliance for phyllodes tumor	6	No	Indeterminant
Compliance with ASBrS or ACR recommendations for stereotactic biopsy reports	6	No	Indeterminant
Time business days from surgeon appointment for cancer to surgery for cancer	6	No	Indeterminant
Percentage of mastectomy patients undergoing reconstruction	6	No	Indeterminant
Cost of perioperative episode of care (affordability)	6	No	Agreement
Patients with cancer diagnosed for core needle biopsy (CNB) for BiRads 4a lesion	6	No	Indeterminant
Patients with cancer diagnosed for CNB for BiRads 4b lesion	6	No	Indeterminant
Patients with cancer diagnosed for CNB for BiRads 4c lesion	6	No	Indeterminant
Patients with cancer diagnosed for CNB for BiRads 5 lesion	6	No	Indeterminant
NCCN guideline compliance for Paget’s disease	6	No	Indeterminant
Surgical-site infection rate (BCS patients)	6	No	Indeterminant
Number of axillary nodes obtained in patients undergoing level 1 or 2 nodal surgery (median)	6	No	Indeterminant
Percentage of DCIS patients undergoing BCS for cancer who do not have axillary surgery	6	No	Indeterminant
Patients with College of American Pathologists (CAP) compliant reporting	5.5	No	Indeterminant
Breast cancer patients presented to interdisciplinary tumor board (real or virtual) before 1st treatment	5.5	No	Indeterminant
Percentage of cancer patients enrolled in clinical trials	5.5	No	Indeterminant
Mastectomy patients with positive SLN who undergo completion of axillary dissection	5.5	No	Indeterminant
Patients with cancer diagnosed on CNB for BiRads 3 lesion	5.5	No	Indeterminant
Patients with unifocal cancer smaller than 3 cm who undergo BCT	5.5	No	Indeterminant
Patients with documentation of pre-op breast size and symmetry	5.5	No	Indeterminant
Clinical stage 0 DCIS patients who do not undergo SLN surgery for BCT	5.5	No	Indeterminant
Patients undergoing level 1 or 2 axillary dissection with ≥15 nodes removed	5.5	No	Indeterminant
Number of SLN’s (median) in patients undergoing SLN procedure	5.5	No	Indeterminant
Breast volume (number of cancer cases per year per surgeon)	5.5	No	Indeterminant
Percentage of cancer patients with documentation of search for clinical trial	5.5	No	Indeterminant
Percentage of breast biopsy pathology requisition forms containing adequate information for pathologist (history, CBE, imaging)	5	No	Agreement
Time from initial cancer surgery to pathology report	5	No	Indeterminant
Patients with documentation of pre-op contralateral breast cancer risk	5	No	Indeterminant
Clinical stage 0 DCIS patients who do not undergo SLN surgery for mastectomy	5	No	Indeterminant
BCT rate (actual and potential)	5	No	Indeterminant
Time business days from abnormal screening mammogram (SM) to office appointment	5	No	Indeterminant
Patients with documentation of needle biopsy results delivered to patients within 48 h	5	No	Indeterminant
BCT-eligible patients offered neoadjuvant treatment	5	No	Agreement
Interval cancers (cancer detected within 1 year after negative US biopsy or stereotactic biopsy)	5	No	Indeterminant
Cosmetic score (measure of cosmesis) after mastectomy, no reconstruction (patient self-assessment)	5	No	Indeterminant
Percentage of cancer patients referred to medical oncology	5	No	Indeterminant
Axillary recurrence rate	5	No	Indeterminant
Patients with NCCN guidelines compliant care for nipple discharge	5	No	Disagreement
Percentage of BCT patients with marker clips placed in lumpectomy cavity to aid radiation oncologist for location of boost dose for radiation	5	No	Indeterminant
Percentage of patients with documentation of arm edema status post-operatively	4.5	No	Indeterminant
Patients undergoing re-operation within 30 days (stratified by case type)	4.5	No	Indeterminant
Patients undergoing re-admission within 30 days (stratified by base type)	4.5	No	Indeterminant
Percentage of BCT patients with oncoplastic procedure performed	4.5	No	Indeterminant
Patients with documentation of gynecologic/sexual side effects of endocrine therapy	4.5	No	Indeterminant
Patients with documentation of gynecologic/sexual changes during follow-up	4.5	No	Indeterminant
Mastectomy patients who undergo immediate intraoperative SLN assessment	4.5	No	Indeterminant
Patients with latragenic injury to adjacent organ, structure (stratified by case type)	4	No	Indeterminant
Percentage of lumpectomy patients with surgeon use of US intraoperatively	4	No	Indeterminant
Patients with documentation of surgical pathology results delivered to patients within 96 h	4	No	Indeterminant
Patients who have “grouped” postoperative appointments (same day, same location with care providers)	4	No	Indeterminant
Percutaneous procedure complications	3.5	No	Indeterminant
Percentage of patients with development of lymphedema of arm after axillary surgery	3.5	No	Indeterminant
Time from initial cancer surgery to pathology report	3	No	Disagreement
Patients with new DVT less than or equal to 30 days post-operatively	3	No	Disagreement
Patients with new PE ≤30 days post-operatively	3	No	Indeterminant
Documentation of use of new NSQIP-generated ACS risk calculator preoperatively	3	No	Indeterminant
Patients with unplanned overnight stay stratified by procedure type	2.5	No	Indeterminant
Sensitivity of immediate intraoperative detection of positive SLN (pathology quality measure)	2.5	No	Agreement
Patients with myocardial infarction ≤30 days postoperatively	2	No	Agreement
Patients with new renal failure ≤30 days postoperatively	2	No	Agreement
Patients with new respiratory failure ≤30 days post-operatively	2	No	Agreement

*ER* estrogen receptor; *PR* progesterone receptor; *HER2* human epidermal growth factor 2; *AJCC* American Joint Committee on Cancer; *NCCN* National Comprehensive Cancer Network; *ADH* Atypical Ductal Hyperplasia; *ALH* Atypical lobular hyperplasia; *FEA* Flat epithelial atypia; *LCIS* Lobular carcinoma in situ; *MRI* magnetic resonance imaging; *SCIP* Surgical care improvment project; *DVT* Deep venous thrombosis; *PE* Pulmonary embolism; *ASBrS* American Society of Breast Surgeons; *ACR* American College of Radiology; *ASTRO* American Society of therapuetic radiation oncologists; *CME* Continuing medical education credits; *DCIS* Ductal carcinoma in situ; *PPV* positive predictive value; *NPV* negative predictive value; *CBE* clinical breast exam; *NSQIP* National Surgical Quality Improvement Program; *ACS* American Cancer Society

^a^Median score 1–9: lowest to highest

^b^Validity: ≥90% of the rankings are in the 7–9 range

^c^Agreement: Based on scoring dispersion (e.g., for a panel of 13, there is “agreement” if >8 rankings are in any 3-point range and disagreement if >3 rankings are 1–3 and 7–9

Italicized text: Final ASBrS QM chosen for CMS quality payment programs

After three rounds of ranking ending in December 2013, nine of the highest ranked measures were “specified” as described and required by CMS^44^ (Table 3). Briefly, exclusions to QM reporting were never included in the performance numerator or denominator. Exceptions were episodes in which performance for a given QM was not met but there was a justifiable reason why that was the case. If so, then the encounter, similar to an exclusion, was not included in the surgeon’s performance rate. If an encounter met performance criteria despite typically meeting exception criteria, the encounter was included in the performance rate. Per CMS rules, each QM was linked to a National Quality Strategy Aim and Domain (Table 3). The QMs also were assigned to a Donabedian category and to one or more of the Institute for Healthcare Improvement’s “triple aims.”^40^,^45^

**Table 3:** American Society of Breast Surgeons Quality Measure Specifications for participation in the Center for Medicaid and Medicare Services Qualified Clinical Data Registry^55^,^56^

QM title	QM name	QM numerator	QM denominator	Exception examples^a^	Exclusion examples^b^	Measure type^c^	NQS domain(s)^d^	IHI triple aim^e^
Needle biopsy	PQRS measure #263: Preoperative diagnosis of breast cancer	No. of patients age ≥18 years undergoing breast cancer operations who had breast cancer diagnosed preoperatively by a minimally invasive biopsy	No. of patients age ≥18 years on date of encounter undergoing breast cancer operations	Lesion too close to implant Patient too obese for stereotactic table Contralateral prophylactic mastectomy Needle performed but identified “high risk” lesion only	Not a breast procedure	Process	Effective clinical care Efficiency and cost reduction	Care experience Per capita cost
Image confirmation	PQRS measure #262: Image confirmation of successful excision of image-localized breast lesion	Patient undergoing excisional biopsy or partial mastectomy of a nonpalpable lesion whose excised breast tissue was evaluated by imaging intraoperatively to confirm successful inclusion of targeted lesion	No. of patients age ≥18 years on date of encounter with nonpalpable, image-detected breast lesion requiring localization of lesion for targeted resection	Target lesion identified intraoperatively by pathology MRI wire localization for lesion occult on mammography and ultrasound	Lesion palpable preoperatively Re-excision surgery for margins Ductal excision without visible lesion on imaging	Process	Patient safety	Care experience
Sentinel node	PQRS measure #264: Sentinel lymph node biopsy for invasive breast cancer	Patients who undergo a sentinel lymph node biopsy procedure	Patients age ≥18 years with clinically node-negative stage 1 or 2 primary invasive breast cancer	Prior nodal surgery Recurrent cancer Limited life expectancy	No preoperative invasive cancer diagnosis Patient has proven axillary metastasis Inflammatory breast cancer	Process	Effective clinical care Safety	Care experience Per capita cost
Hereditary assessment	ASBS 1: Surgeon assessment for hereditary cause of breast cancer	No. of breast cancer patients with newly diagnosed invasive and DCIS seen by surgeon who undergo risk assessment for a hereditary cause of breast cancer	No.of newly diagnosed invasive and DCIS breast cancer patients seen by surgeon and who undergo surgery	Patient was adopted Family history not obtainable for any specific reason	LCIS patients Patient does not have breast cancer or patient does not undergo surgery	Process	Effective clinical care Community and population health (e.g., screening for germline mutation in family members)	Care experience Population health
Surgical-site infection	ASBS 2: Surgical-site infection and cellulitis after breast and/or axillary surgery	No. of patients age ≥18 years who experience an SSI or cellulitis within 30 days after undergoing a breast and/or an axillary operation	No. of patients age ≥18 years on date of encounter undergoing a breast and/or axillary operation	None	Patient did not undergo breast or axillary surgery	Outcome	Person and caregiver-centered experience and outcomes	Care experience Per capita cost
Specimen orientation	ASBS 3: Specimen orientation for partial mastectomy or excisional breast biopsy	No. of patients age ≥18 years undergoing a therapeutic breast surgical procedure considered an initial partial mastectomy or “lumpectomy” for a diagnosed cancer or an excisional biopsy for a lesion that is not clearly benign based on previous biopsy or clinical and radiographic criteria with surgical specimens properly oriented for pathologic analysis such that six margins can be identified	No. of patients age ≥18 years undergoing a therapeutic breast surgical procedure considered an initial partial mastectomy or “lumpectomy” for a diagnosis of cancer or an excisional biopsy for a lesion that is not clearly benign based on previous biopsy or clinical and radiographic criteria	Clinical and imaging findings suggest benign lesion (e.g., fibroadenoma)	Patients who had total mastectomy (all types)	Process	Communication and care coordination	Care experience
Antibiotic choice	ASBS 5: Perioperative care: Selection of prophylactic antibiotics: first- or second-generation cephalosporin (modified for breast from PQRS measure #21)	Surgical patients age ≥18 years undergoing procedures with indications for a first- or second- generation cephalosporin prophylactic antibiotic who had an order for a first- or second -generation cephalosporin for antimicrobial prophylaxis	All surgical patients age ≥18 years undergoing procedures with the indications for a first- or second -generation cephalosporin prophylactic antibiotic	Patient allergic to cephalosporins	Not a breast procedure	Process	Patient safety	Care experience Per capita cost
Antibiotic duration	ASBS 6: Perioperative care: Discontinuation of prophylactic parenteral antibiotics (modified for breast from PQRS measure #22)	Noncardiac surgical patients who have an order for discontinuation of prophylactic parenteral antibiotics within 24 h after surgical end time	All noncardiac surgical patients age ≥18 years undergoing procedures with the indications for prophylactic parenteral antibiotics and who received a prophylactic parenteral antibiotic	Antibiotic *not* discontinued: ordered by plastic surgeon for expander or implant insertion	Not a breast procedure	Process	Patient safety	Care experience Per capita cost
Mastectomy reoperation	Unplanned 30-day re-operation rate after mastectomy	Patients undergoing mastectomy who do not require an unplanned secondary breast or axillary operation within 30 days after the initial procedure	Patients undergoing uni- or bilateral mastectomy as their initial proscedure for breast cancer or prophylaxis.	Patients who have a contralateral breast reoperation attributed to plastic surgeon for a complication in a breast not operated on by the breast surgeon Patients with autologous flap necrosis attributed to plastic surgeon	Patient underwent lumpectomy as his or her initial operation	Outcome	Patient safety Efficiency and cost reduction	Care experience Per capita cost

Specifications^55^,^56^

*QM* quality measure; *NQS* National Quality Strategy; *IHI* Institute for Healthcare Improvement; *PQRS* Physicians Quality Reporting Service; *MRI* magnetic resonance imaging; *ASBS* American Society of Breast Surgeons; *LCIS* Lobular carcinoma in situ; *DCIS* ductal carcinoma in situ; *SSI* surgical site infection

^a^Exceptions mean the patient encounter is included only in the numerator and denominator if “performance was met.”

^b^Exclusions mean the patient encounter is never included in the numerator or denominator

^c^Donabedian domain^40^

^d^National Quality Strategy domain^41^

^e^Institute for Healthcare Improvement Triple Aim^45^

Each of our QMs underwent vetting in our electronic patient registry (Mastery) by a workgroup before submission to CMS. During this surveillance, a QM was modified, retired, or advanced to the QCDR program based on member input and ASBrS Executive Committee decisions.

### Patient Encounters

To calculate the total number of provider-patient-measure encounters captured in Mastery, we summed the total reports for each individual QM for all study years and all providers who entered data.

### Benchmarking

**Fig. 1:**
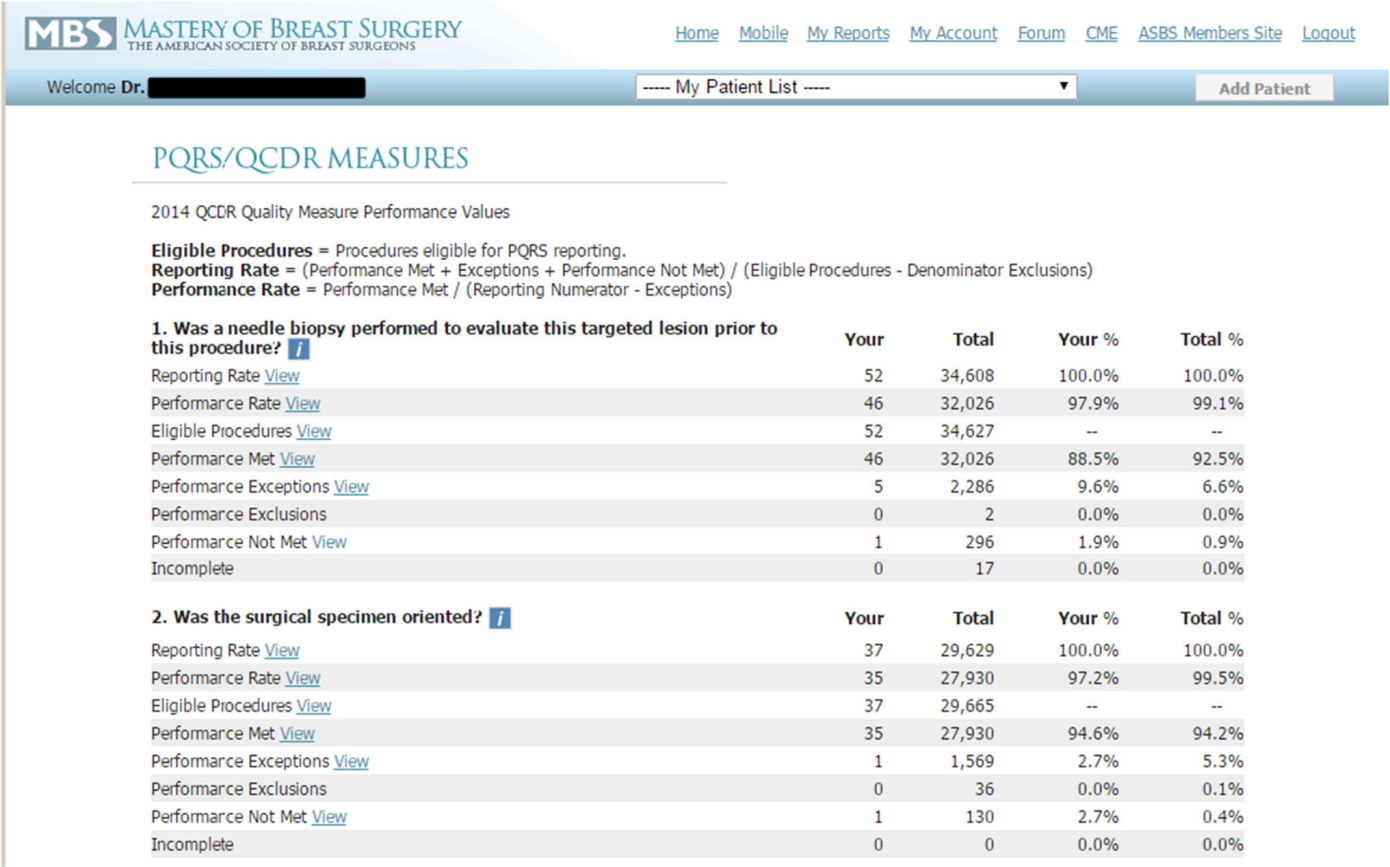
Example of real-time peer performance comparison after surgeon entry of quality measures

Each surgeon who entered data into Mastery was able to compare his or her up-to-date performance with the aggregate performance of all other participating surgeons (Fig. 1). The surgeons were not able to access the performance metrics of any other named surgeon or facility.

### Data Validation

In compliance with CMS rules, a data validation strategy was performed annually. A blinded random selection of at least 3% of QCDR surgeon participants was conducted. After surgeons were selected for review, the ASBrS requested that they send the ASBrS electronic and/or paper records to verify that their office/hospital records supported the performance “met” and “not met” categories that they had previously reported to the ASBrS via the Mastery registry.

## Results

### Hierarchical Order and CMS QCDR Choices

The median ranking scores for 144 potential QMs ranged from 2 to 9 (Table 2). The nine QMs chosen and their ranking scores were appropriate use of preoperative needle biopsy (9.0), sentinel node surgery (9.0), specimen imaging (9.0), specimen orientation (9.0), hereditary assessment (7.0), mastectomy reoperation rate (7.0), preoperative antibiotics (7.0), antibiotic duration (7.0), and surgical-site infection (SSI) (6.0). The specifications for these QMs are presented in Table 3. The mastectomy reoperation rate and SSI are outcome measures, whereas the remainder are process of care measures.

### QM Encounters Captured

A total of 1,286,011 unique provider-patient-measure encounters were captured in Mastery during 2011–2015 for the nine QCDR QMs. Performance metrics and trends for each QM are reported separately.

### Data Validation

The QM reporting rate of inaccuracy by surgeons participating in the 2016 QCDR data validation study of the 2015 Mastery data files was 0.82% (27 errors in 3285 audited patient-measure encounters). Subsequent reconciliation of discordance between surgeon QM reporting and patient clinical data occurred by communication between the ASBrS and the reporting provider.

### CMS Acceptance and Public Transparency

The Center for Medicare and Medicaid Services accepted the ASBrS QM submitted to them for PQRS participation in 2010–2013 and for QCDR in 2014–2016. In 2016, they discontinued the specimen orientation measure for future reporting and recommended further review of the mastectomy reoperation rate measure. Public reporting of 2015 individual surgeon QCDR data was posted in 2016 on the ASBrS website.

### Security

To our knowledge, no breaches have occurred with any surgeon-user of Mastery identifying the performance of any other surgeon or the identity of any other surgeon’s patients. In addition, no breaches by external sources have occurred within the site or during transmission of data to CMS.

## Discussion

### Modified Delphi Ranking of QM

To provide relevant QM for our members, the PSQC of the ASBrS completed a hierarchal ranking of more than 100 candidate measures and narrowed the collection of QMs to fewer than a dozen using accepted methods.^42^,^43^ Although not reported here, the same process was used annually to identify new candidate QMs from 2014 to 2017 for future quality payment programs and to develop measures for the Choosing Wisely campaign.^46^ Based on our experience, we recommend its use for others wanting to prioritize longer lists of potential QM domains into shorter lists. These lists are iterative, allowing potential measures to be added anytime, such as after the publication of clinical trials or after new evidence-based guidelines are developed for better care. In addition, with the modified Delphi ranking process, decisions are made by groups, not individuals.

### After Ranking, What Next?

Of the nine QMs selected for submission to CMS, only four had the highest possible ranking score. The reasons for not selecting some highly ranked domains of care included but were not limited to the following concerns. Some QMs were already being used by other organizations or were best assessed at the institutional, not the surgeon, level, such as the use of radiation after mastectomy for node-positive patients.^32^–^36^ Other highly ranked measures, such as “adequate history,” were not selected because they were considered standard of care.

Contralateral prophylactic mastectomy rates, a contemporary topic of much interest, was not included in our original ranking, and breast-conserving therapy (BCT) was not ranked high due to our concern that both were more a reflection of patient preferences and of regional and cultural norms than of surgeon quality. A lumpectomy reoperation QM was ranked high (7.5), but was not chosen due to disagreement within the ASBrS whether to brand this a quality measure.^47^,^48^ In some cases, QMs with lower scores were selected for use for specific reasons. For example, by CMS rules, two QMs for a QCDR must be “outcome” measures, but all our highest ranked measures were “process of care” measures.

There was occasional overlap between our QM and those of other organizations.^21^,^32^–^39^ In these cases, we aimed to harmonize, not compete with existing measures. For example, a patient with an unplanned reoperation after mastectomy would be classified similarly in both the National Surgical Quality Improvement Program (NSQIP) and our program. In contrast to NSQIP, we classified a patient with postoperative cellulitis as having an SSI. Because excluding cellulitis as an SSI event has been estimated to reduce breast SSI rates threefold, adoption of the NSQIP definition would underestimate the SSI burden to breast patients and could limit improvement initiatives.^49^

### Governance

Ranking and specifying QMs is arduous. Consensus is possible; unanimous agreement is rare. Therefore, a governance structure is necessary to reconcile differences of opinion. In our society, the PSQC solicits, ranks, and specifies QMs. A workgroup vets them for clarity and workability. In doing so, the workgroup may recommend changes. The ASBrS Executive Committee reconciles disputes and makes final decisions .

### Reporting Volume

Our measurement program was successful, capturing more than 1 million provider-patient-measure encounters. On the other hand, our member participation rate was less than 20%. By member survey (not reported here), the most common reason for not participating was “burden of reporting.”

### Benchmarking

 “Benchmarking” is a term used most often as a synonym for peer comparison, and many programs purport to provide it.^25^ In actuality, benchmarking is a method for improving quality and one of nine levers endorsed by the National Quality Strategy to upgrade performance.^21^,^23^,^30^,^50^ Believing in this concept, the ASBrS and many other professional societies built patient registries that provided benchmarking.^21^,^25^,^32^–^35^ In contradistinction, the term “benchmark” refers to a point of reference for comparison. Thus, a performance benchmark can have many different meanings, ranging from a minimal quality threshold to a standard for superlative performance.^24^,^36^

## Program strengths

Our patient registry was designed to collect specialty-specific QMs as an alternative to adopting existing general surgical and cross-cutting measures. Cross-cutting measures, such as those that audit medicine reconciliation or care coordination, are important but do not advance specialty-specific practice. Furthermore, breast-specific measures lessen potential bias in the comparison of providers who have variable proportions of their practice devoted to the breast. Because alimentary tract, vascular, and trauma operations tend to have higher morbidity and mortality event rates than breast operations, general surgeons performing many non-breast operations are not penalized in our program for a case mix that includes these higher-risk patients. In other words, nonspecialized general surgeons who want to demonstrate their expertise in breast surgery can do so by peer comparison with surgeons who have similar case types in our program. In addition, a condition-specific program with public transparency allows patients to make more informed choices regarding their destination for care. In 2016, individual provider report-carding for our participating surgeons began on the “physician-compare” website.^51^

Another strength of an organ-specific registry is that it affords an opportunity for quick Plan-Do-Study-Act (PDSA) cycles because personal and aggregate performance are updated continuously. Thus action plans can be driven by subspecialty-specific data, not limited to expert opinion or claims data. For example, a national consensus conference was convened, in part, due to an interrogation of our registry that identified wide variability of ASBrS member surgeon reoperation rates after lumpectomy.^52^,^53^ Other program strengths are listed in Table 4.

**Table 4:** Strengths and limitations of the American Society of Breast Surgeons quality measurement program

*Strengths*
Specialty measures and their specifications developed by surgeons
Justifiable “exceptions” to not meeting performance defined by surgeons
Real-time surgeon data entry lessens recall bias, abstractor error, and misclassification of attribution for not meeting a performance requirement
Real-time peer performance comparison
Large sample size of patient-measure encounters (>1,000,000) for comparisons
General surgeons able to compare breast surgical performance to breast-specialty surgeons
Low level of erroneous reporting based on audits
Participation satisfies American Board of Surgery Maintenance of Certification Part 4
Public transparency of individual surgeon performance in 2015 on the American Society of Breast Surgeons (ASBrS) website in 2016
Capability to use the program for “plan-do-study-act” cycles^52^,^53^
No participation fee for members before 2016^a^
*Limitations*
Peer performance comparison not yet risk-adjusted
Unknown rate of nonconsecutive patient data entry
No significant patient or payer input into quality measure list or ranking to reflect their preferences and values^54^
Unknown rate of surgeon “dropout” due to their perception of poor performance

^a^$100.00 began 2016

## Study Limitations

Although risk-adjusted peer comparisons are planned, to date, we are not providing them. In addition, only the surgeons who participate with CMS through our QCDR sign an “attestation” statement that they will enter “consecutive patients,” and no current method is available for cross-checking the Mastery case log with facility case logs for completeness. Recognizing that nonconsecutive case entry (by non-QCDR surgeons) could alter surgeon performance rates, falsely elevating them, one investigation of Mastery compared the performance of a single quality indicator between QCDR- and non–QCDR-participating surgeons.^52^ Performance did not differ, but this analysis has not been performed for any of the QMs described in this report. Surgeons also can elect to opt out of reporting QMs at any time. The percentage of surgeons who do so due to their perception of comparatively poor performance is unknown. If significant, this self-selected removal from the aggregate data would confound overall performance assessment, falsely elevating it.

Another limitation is our development of QMs by surgeons with minimal patient input and no payer input. As a result, we cannot rule out that these other stakeholders may have a perception of the quality of care delivered to them that differs from our perception. For example, patients might rank timeliness of care higher than we did, and payers of care might rank reoperations the highest, given its association with cost of care. We may not even be measuring some domains of care that are most important to patients because we did not uniformly query their values and preferences upfront during program development, as recommended by others.^2^,^54^ See Table 4 for other limitations.

## Conclusion

 In summary, the ASBrS built a patient registry to audit condition-specific measures of breast surgical quality and subsequently provided peer comparison at the individual provider level, hoping to improve national performance. In 2016, we provided public transparency of the 2015 performance reported by our surgeon participants.^55^,^56^ In doing so, we have become stewards, not bystanders, accepting the responsibility to improve patient care. We successfully captured more than 1 million patient-measure encounters, participated in CMS programs designed to link reimbursement to performance, and provided our surgeons with a method for satisfying American Board of Surgery Maintenance of Certification requirements. As public and private payers of care introduce new incentivized reimbursement programs, we are well prepared to participate with our “tested” breast-specific QMs.
